# Plasma circulating microRNAs in patients with stable coronary artery disease – Impact of different cardiovascular risk profiles and glomerular filtration rates

**Published:** 2021-04-16

**Authors:** Karlis Trusinskis, Maris Lapsovs, Sandra Paeglite, Evija Knoka, Laima Caunite, Mairita Mazule, Ieva Briede, Sanda Jegere, Indulis Kumsars, Inga Narbute, Ilze Konrade, Andrejs Erglis, Aivars Lejnieks

**Affiliations:** ^1^Latvian Centre of Cardiology, Pauls Stradins Clinical University Hospital, Riga, LV-1002, Latvia; ^2^Department of Internal Diseases, Riga Stradins University, Riga, LV-1007, Latvia; ^3^Faculty of Medicine, University of Latvia, Riga, LV-1050, Latvia; ^4^Department of Endocrinology and Internal Medicine, Riga East Clinical University Hospital, Riga, LV-1038, Latvia

**Keywords:** coronary artery disease, microRNA-126, microRNA-145, microRNA-155, SYNTAX score

## Abstract

**Background and Aim:**

Plasma circulating microRNA (miRNA)-126, -145, and -155 are associated with vascular remodeling, atherosclerotic lesion formation, and plaque vulnerability. In this study, we evaluated the levels of plasma circulating miRNAs in patients with stable coronary artery disease (CAD), different cardiovascular risk profiles, and different glomerular filtration rates (GFR).

**Methods and Results:**

Forty patients with stable CAD admitted for elective percutaneous coronary intervention (PCI) were enrolled in a prospective study. Before PCI, fasting blood samples were obtained to evaluate clinical parameters and miRNA-126 and miRNA-155 expression. The GFR was calculated by the MDRD and CKD-EPI formulas, and the severity of CAD was calculated according to the SYNTAX score. All these parameters were correlated with miRNAs. The association between miRNA levels and clinical characteristics was evaluated. The expression of miRNA-126 positively correlated with a higher SYNTAX score (r = 0.337; p=0.034); however, no significant correlations between miR-126, GFR, and clinical characteristics were observed. Higher plasma levels of miRNA-155 correlated with increased levels of triglycerides (r = 0.317; P = 0.049), C-peptide (r = 0.452; P = 0.011), and the HOMA index (r = 0.447; P = 0.012) and a higher body mass index (BMI) (r = 0.385; P = 0.015). GFR and miRNA-155 (MDRD – Rho=0.353; P = 0.027. CKD-EPI – Rho=0.357; P = 0.026) were found to have a moderate correlation, although miRNA-155 had no correlation with the SYNTAX score.

**Conclusion:**

Plasma circulating miRNA-126 levels were increased in patients with severe atherosclerosis as determined by the SYNTAX score. Elevated miRNA-155 expression was observed in patients with Stage 1 GFR but was lower in patients with Stages 2 and 3 GFR. Plasma circulating miRNA-155 had positive correlations with higher levels of BMI, HOMA index, C-peptide, and triglycerides.

**Relevance for Patients:**

Although further investigations are needed to confirm the role of miRNA-155 and miRNA-126, they may serve as potential biomarkers detecting severity of CAD, lowering of kidney function and metabolic syndrome.

## 1. Introduction

MicroRNAs (miRNAs) are a class of small, single-stranded, non-coding RNA molecules. Various studies have shown that these units have an important role in both physiological and pathological processes. Some researchers are already targeting their potential diagnostic possibilities in diseases such as atherosclerosis due to valuable impacts on the cell cycle and differentiation, immunomodulation, and fat metabolism [1-4]. The specific vascular miRNAs-126, -145, and -155 have attracted interest among cardiologists due to their involvement in atherogenesis through regulation of the inflammatory response in endothelial cells (ECs), activation of M1/M2 macrophages, and proliferation of vascular smooth muscle cells (VSMCs) [4,5].

miRNA-126 is mainly secreted in vascular ECs. The paracrine effects of these molecules are the regulation of VSMC gene expression and cellular functions such as VSMC growth and differentiation [6]. Through this pathway, miRNA-126 plays an important role in EC and VSMC interactions by modulating vascular homeostasis and decreasing atherosclerotic processes. Higher levels of miRNA-126 in vessel walls are associated with intimal VSMC proliferation, higher collagen content, and the regulation of efferocytosis, consequently reducing apoptotic cells and resulting in a more stable plaque phenotype with increased fibrous collagen saturation [4]. miRNA-145 is mostly expressed in VSMCs. Compared to other plasma circulating miRNAs, it is the most highly secreted miRNA in arteries. Using several pathways, circulating levels of miRNA-145 can lead to local overexpression. This upregulation can not only limit plaque burden but also reduce inflammation and increase plaque stability by promoting the VSMC contractile phenotype and stabilizing the fibrous cap in more advanced plaques [7]. However, increased circulating miRNA-145 levels are also associated with decreased VSMC proliferation and reduced ATP-binding cassette transporter (ABCA1) expression and therefore decreased cholesterol efflux in cells [4]. miRNA-155 is specifically expressed in pro-inflammatory macrophages and, in the case of atherosclerosis, promotes foam cell formation through the miRNA-155-HBP1 signaling pathway. Interestingly, the expression of miRNA-155 is significantly higher in CD14^+^ monocytes in patients with coronary artery disease (CAD) than in healthy individuals [4]. It stimulates inflammatory processes by promoting monocyte recruitment to atherosclerotic plaques and is a viable target to promote M2 macrophage polarization or reprogram M1 macrophages, which reduces chronic inflammation and is thus a key point in atherosclerosis regression [8].

Cardiovascular disease (CVD) risk factors include a wide range of different lifestyle and genetic aspects, which have impacts on cardiovascular events and are connected with plasma circulating miRNAs [9,10]. Moreover, the development of CVD can be affected differently by each of these factors. For example, in chronic kidney disease (CKD), even a mild-to-moderate reduction in the glomerular filtration rate (GFR) promotes the development of CAD and even more severe disease courses. In this case, disease development is associated with plasma circulating miRNA levels [11-13]. Although the impact of plasma circulating miRNAs on atherogenesis has been studied, this novel field lacks answers about the interconnection between these molecules in patients with CAD with different cardiovascular risk factors [10]. The aim of the current study was to determine the connection among plasma circulating miRNA-126, -145, and -155 expression, the severity of CAD assessed by the SYNTAX score, different estimated GFR (eGFR), and cardiovascular risk profiles [5].

## 2. Materials and Methods

### 2.1. Patients

A total of 40 patients with stable CAD undergoing elective percutaneous coronary intervention (PCI) from August 2016 until September 2018 were enrolled in a prospective, single high-volume PCI center study. The study was conducted in accordance with the ethical principles of the Helsinki Declaration (WMA, 2013). Patient inclusion criteria were patient age ≥18 years, stable CAD, and at least one risk factor: Arterial hypertension, dyslipidemia, smoking, body mass index (BMI) >25 kg/m^2^, and eGFR 89–30 ml/min/1.73 m^2^. Patients were excluded from the study if they met any of the following exclusion criteria: Life expectancy <2 years, admitted due to acute coronary syndrome, severe blood vessel calcification, previous PCI in the target segment, chronic heart failure (NYHA functional class III-IV), diabetes mellitus or glucose lowering therapy, and Stage IV-V CKD. All participants gave their written informed consent. A standard fasting venous blood sample was taken to determine HbA1c, glucose, serum total cholesterol, high-density lipoprotein cholesterol (HDL-C), low-density lipoprotein cholesterol (LDL-C), C-peptide, triglyceride, and creatinine levels. The HOMA index was estimated using the Oxford University calculator, which takes into account C-peptide and glucose. All patients received statin therapy before and during the study.

### 2.2. Calculation of miRNA

Fasting venous blood samples in EDTA-containing tubes were taken before PCI. To isolate plasma, we used rapid centrifugation for 20 min at 4°C. The supernatant was stored at –80°C in RNAse/DNAse-free tubes. The MiRNeasy Serum/Plasma kit protocol (Qiagen, Valencia, CA) was used to obtain total RNA containing miRNAs. Before the RNA purification process, cel-miRNA-39 was added as a spike-in control to avoid differences in template quality and confirm the efficiency of the reverse transcription reaction and to normalize the relative Ct values of miRNA-126, miRNA-145, and miRNA-155 in subsequent data analyses. Cel-MiRNA-39 was used as a spike-in control since it has no mammalian homologue [14]. A TaqMan miRNAs Reverse Transcription kit (Applied Biosystems, CA, USA) was used to perform reverse transcription. TaqMan miRNA assay kits (Applied Biosystems) were used for miRNA amplification, and real-time polymerase chain reaction (RT-PCR) was performed to detect miRNA-126, miRNA-145, and miRNA-155 expression. For further data analysis, the relative expression levels of miRNAs were calculated using the comparative delta *Ct* (threshold cycle number) method (2^−DCT^) implemented in the RT-PCR System software. The relative *Ct* for cel-miRNA-39 was used as a control in the normalization of miRNA-126, miRNA-145, and miRNA-155 *Ct*.

### 2.3. GFR formula

The GFR was estimated using the abbreviated Modification of Diet in Renal Disease (MDRD) and CKD Epidemiology Collaboration 2009 (CKD-EPI) formulas. Patients were divided into five CKD stages according to their eGFR.

*SYNTAX score*: SYNTAX or Synergy between PCI with TAXUS and Cardiac Surgery is an accepted tool for the determination of CAD severity. All visible stenoses >50% in coronary arteries with diameters >1.5 mm were taken into account using the SYNTAX score calculator. It has already been proven that the SYNTAX score can be used to assess revascularization prognosis [5]. Based on revascularization prognosis, the SYNTAX score was divided into three groups: Low, 0–22; intermediate, 23–32; and high ≥33. Due to the low sample size with a high SYNTAX score, intermediate and high SYNTAX scores were combined into one group.

### 2.4. Statistical analysis

Data are reported as median values. Correlations between miRNAs, SYNTAX score, eGFR, and other biochemical parameters were compared using Spearman’s correlation analysis for non-parametric data and Pearson’s correlation for parametric data (Table 1, laboratory parameters). The threshold for statistical significance was set to *P* < 0.05. Statistical analyses were performed using SPSS version 23 software.

**Table 1:** Association between laboratory parameters and miRNA-126, -145, and -155 expressions

Pearson correlation	miRNA-126		miRNA-145		miRNA-155	
		
	*r*	*P*	*r*	*P*	*r*	*P*
Total cholesterol	0.158	0.329	-0.121	0.455	0.126	0.443
LDL-C	0.12	0.462	-0.176	0.278	-0.169	0.304
HDL-C	0.202	0.212	-0.154	0.342	0.113	0.495
Triglycerides	0.199	0.217	0.252	0.117	0.317	0.049
BMI	0.079	0.628	0.026	0.874	0.385	0.015
C-peptide	-0.005	0.980	-0.224	0.225	0.452	0.011
HbA1c	-0.098	0.553	-0.2	0.223	0.102	0.541
Fasting blood glucose	0.018	0.912	-0.186	0.257	0.285	0.079
HOMA index	0.005	0.978	-0.234	0.205	0.447	0.012

*BMI: Body mass index, HDL-C: High-density lipoprotein cholesterol, LDL-C: Low-density lipoprotein cholesterol, HbA1c: Glycated hemoglobin.

## 3. Results

The main patient characteristics are shown in Table 2.

**Table 2:** Baseline patient characteristics with low and medium to high SYNTAX scores

Variables	CAD by SYNTAX score	
SYNTAX score (number of patients)	≤22 (*n*=35)	>22 (*n*=5)
Age (years), median	60	63
BMI kg/m^2^ median	30.02	29.74
Current smoking, %	37.1	60
Hypertension, %	97.1	100
Atrial fibrillation, %	20	20
Peripheral artery disease, %	11.4	0
Previous PCI, %	60	40
Chronic heart disease, %	73.5	40
Previous stroke, %	8.6	0
Previous myocardial infarction, %	40	80
Total cholesterol (mmol/L), median	3.81	4.3
LDL-C (mmol/L), median	1.93	1.69
LDL-C<1.8 mmol/L, %	42.8	60
HDL-C (mmol/L), median	1.28	1.08
Triglycerides (mmol/L), median	1.31	2.19
Fasting blood glucose (mmol/L), median	5.2	4.9
HbA1c (%), median	5.9	5.8
C-peptide (ng/mL), median	2.19	2.53
HOMA index	1.605	1.840
Creatinine (mmol/L), median	74	71
Estimated glomerular filtration rate		
MDRD (ml/min/1.73 m^2^), median	84	97
CKD-EPI (ml/min/1.73 m^2^), median	90	94
Estimated glomerular filtration rate stages		
MDRD (ml/min/1.73 m^2^), I/II-III stages (n)	I – 14	I – 3
	II – 18	II – 1
	III – 3	III – 1
CKD-EPI (ml/min/1.73 m^2^), I/II-III stages (n)	I – 18	I – 3
	II – 14	II – 1
	III – 3	III – 1
Relative miRNA expression, median		
miRNA-126	3.61	4.27
miRNA-145	6.11	7.09
miRNA-155	7.26	6.83

*BMI: Body mass index; HDL-C: High-density lipoprotein cholesterol; LDL-C: Low-density lipoprotein cholesterol; LDL-C<1.8 mmol/L – European Society of Cardiology (ESC) defined target level of LDL-C for high-risk patients<1.8 mmol/L (according to ESC guidelines, which were in effect at the time of the research) [15]; PCI: Percutaneous coronary intervention; SD: Standard deviation

Higher levels of miRNA-155 correlated with increased levels of triglycerides, C-peptide, HOMA index, and higher BMI (Table 1).

**Figure 1:**
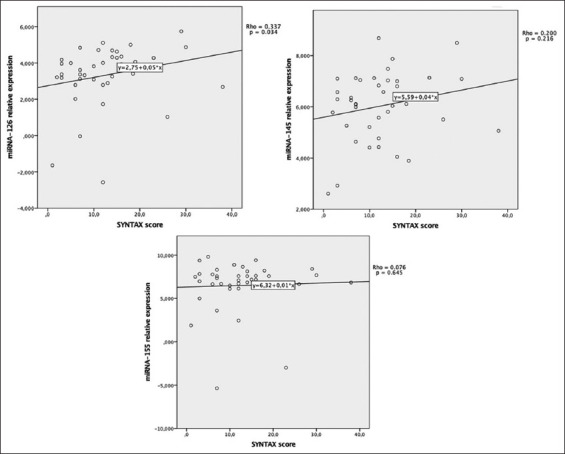
Correlations between plasma circulating miRNA expression and the SYNTAX score

SYNTAX score results were compared with plasma circulating miRNAs (Figure 1). Higher levels of circulating miRNA-126 positively correlated with higher SYNTAX scores (*r* = 0.337; *P* = 0.034).

**Figure 2:**
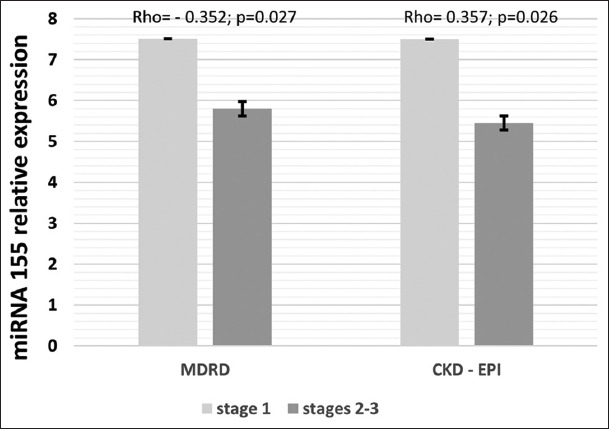
Association between plasma circulating miRNA-155 levels and different stages of chronic kidney disease according to the MDRD and CKD-EPI (2009) formulas.* Stage 1: Normal estimated glomerular filtration rate (>90 ml/min), Stages 2-3: Pathological estimated glomerular filtration rate (<90 ml/min). Patients (n): MDRD – Stage 1 = 17, Stages 2–3 = 23; CKD-EPI – Stage 1 =- 21, Stages 2–3 = 19.

The mean eGFR calculated by the MDRD and CKD-EPI methods had a positive correlation with higher levels of plasma circulating miRNA-155 (Table 3). Decreasing levels of miRNA-155 in different eGFR stages are provided in Figure 2.

**Table 3:** Association between miRNA-126, -145, and -155 expression and the estimated glomerular filtration rate calculated by the MDRD and CKD-EPI (2009) formulas

Spearman’s rank correlation	miRNA-126		miRNA-145		miRNA-155	
		
	*Rho*	*P*	*Rho*	*P*	*Rho*	*P*
eGFR MDRD	-0.048	0.767	-0.026	0.874	0.353	0.027
eGFR CKD-EPI	-0.077	0.638	-0.014	0.934	0.357	0.026

* eGFR: Estimated glomerular filtration rate

## 4. Discussion

The main results of our study are as follows: miRNA-126 correlates with severe CAD assessed by the SYNTAX score; the levels of miRNA-155 correlate with higher levels of eGFR by both the CKD-EPI and MDRD formulas as well as with higher BMI, C-peptide, HOMA index, and triglycerides.

Large-scale evidence shows that plasma circulating miRNA-126 plays an important role in the initiation of vascular tissue repair or angiogenesis and stabilization of already formed plaques [16]. Our study results show an association between higher levels of miRNA-126 and increased SYNTAX scores. These findings impede the clarification of the exact role of miRNA-126 and enhance the conflict among other studies in this field. Fichtlscherer *et al*. (2010) reported that miRNA-126 levels in severe CAD cases were lower than those in control groups [17]. In contrast, Sun *et al*. (2012) and Li *et al*. (2016) reported no significant difference in the expression of plasma circulating miRNA-126 in patients with CAD with low SYNTAX scores and control subjects, which is in line with our findings (median SYNTAX score was 12) [18,19]. These studies raise questions about the different informative values of plasma circulating miRNA-126 depending on the severity of CAD. Therefore, it could be suggested that in patients with mild CAD, plasma circulating miRNA-126 expression could be affected by the cumulative effect of different cardiovascular risk factors and not by atherosclerosis severity. For example, Sun *et al*. (2012) found higher levels of miRNA-126 in patients with increased LDL levels without angiographically significant CAD. It has been suggested that the levels of miRNA-126 may reflect a compensatory response to inflammation under a hyperlipidemic background; however, the pathways by which LDL can affect miRNA levels and vice versa remain unclear [19]. Li *et al*. (2016) reported that the levels of plasma circulating miRNA-126 were influenced by cardiovascular risk factors, including ageing, diabetes mellitus, and hyperlipidemia [18]. In addition, different plaque types of diseased vessels might be responsible for the contrary results among studies in this field. We suggest that the levels of miRNAs might vary not only because of diffuse atherosclerosis, which is a major criterion in the SYNTAX score, but also due to atherosclerotic activity and plaque type. The previous studies reported that one of the dysfunctional roles of ECs in atherosclerosis is upregulating the expression of chemokines/adhesion molecules [20]. Our previous center research already showed that miRNA-126 levels are increased in patients with higher necrotic tissue content in coronary arteries compared to patients with other plaque types [21]. This finding is in line with results that showed the role of miRNAs in plaque stabilization. Zernecke *et al*. (2009) found that miRNA-126 carries vascular protection through CXCL12, where EC apoptotic bodies stimulate this pathway and increase plasma circulating miRNA-126 levels [22]. Our results show that the value of circulating miRNA-126 in plasma as an isolated biomarker is limited, mostly due to its dependence on other physiological processes. Further studies are needed to prove its potential prognostic value in cooperation with other miRNA molecules.

In the present study, circulating levels of miRNA-145 and miRNA-155 showed no correlation with severity of CAD assessed by SYNTAX score. Maitras *et al*. (2017) described the increased expression of local miRNA-145 in vulnerable carotid plaques. Higher circulating miRNA-145 levels were also observed in patients with ischemic stroke. These findings suggest a different impact of plasma circulating and locally expressed miRNA-145 in carotid and coronary plaques. Both atheroprotective and atherogenic effects of miRNA-155 have been observed in studies with animal models [23]. Perhaps, miRNA-155 impact on atherosclerosis is competitive with other metabolic factors like the ones discussed below.

Our study indicates that higher miRNA-155 expression positively correlates with increased BMI, HOMA index, C-peptide, and triglycerides. This observation suggests that miRNA-155 is involved not only in inflammation regulation in atherosclerosis but also in metabolic processes. miRNA-155 mainly promotes its role through regulation of macrophage differentiation. Overexpression of miRNA-155 shifts macrophages toward the pro-inflammatory M1 type but inhibits macrophages polarization toward the anti-inflammatory M2 type [24]. Longo *et al*. (2019) and Karkeni *et al*. (2017) reported that pro-inflammatory M1-type macrophages upregulate miRNA-155 expression and are strongly linked with obesity and inflammation, leading to insulin resistance [25,26]. It has been observed that pro-inflammatory cytokines can stimulate insulin resistance by disrupting insulin receptor substrate (IRS) protein signaling and activate the NF-kB (nuclear factor kappa-light-chain-enhancer of activated B cells) and JNK (c-Jun NH2-terminal kinase) signaling pathways, which affects insulin resistance [27]. It is well known that obesity generates low-grade local and systemic inflammation, leading to the enhanced expression of pro-inflammatory cytokines [25]. Chronic inflammation induces pro-inflammatory M1 macrophage upregulation in adipose tissue, thus increasing plasma circulating miRNA-155 expression [5]. It has been suggested that M1 macrophages inhibit adipocyte differentiation by secreting pro-inflammatory cytokines, which leads to adipocyte hypertrophy and ectopic storage of triglycerides [28]. In our cohort, systemic inflammation contributed more to insulin resistance than to the development of atherosclerosis. Further investigations are needed to confirm the role of miRNA-155 and regulatory mechanisms in obesity, insulin resistance, and lipoprotein metabolism and the impact of these processes on atherosclerosis development.

Our data demonstrated downregulation of miRNA-155 by the slightly reduced eGFR in Stage 2-3 CKD compared with Stage 1 CKD, and these results are consistent with the previous studies showing a significant reduction in circulating miRNAs in patients with CKD [29]. This result is explained by the pathophysiological pathways of angiotensin II, especially angiotensin receptor subtype 1 (AT1R), in cardiovascular and renal system diseases. It has been discovered that plasma circulating miRNA-155 can reduce the pro-inflammatory activity of AT1R, thereby reducing the inflammatory/oxidative response, hypertension, endothelial dysfunction, and vascular remodeling [5,30]. Chen *et al*. (2013) reported that overexpression of miRNA-155 suppresses AT1R, thus inhibiting cell proliferation. Consequently, low expression of miRNA-155 may be a causative factor in the proliferative VSMC state observed in CKD and may even play a role in CVD and renal fibrosis [31]. Neal *et al*. (2011) and Chen *et al*. (2013) similarly observed that the concentration of circulating plasma miRNA-155 is reduced in patients with impaired kidney function [31,32]. More studies should be performed to investigate the underlying mechanisms of the miRNA-155 interaction with kidney function.

We did not observe correlation between miRNA-126 and mild-to-moderate CKD. Fourdinier *et al*. (2019) studied 598 CKD patients (Stage 1–5 including patients on dialysis) and 31 healthy controls. He showed a strong association between miRNA-126 and eGFR, but not with all-cause mortality, cardiovascular, and renal events [33]. Differences in severity of CKD of included patients along with a small sample size of our study are possible explanations for these contradicting results.

### 4.1. Limitations

There are several limitations to this study. First, there was a small sample size, lacks a control group and control to exclude RNA degradation. Second, we did not take into consideration kidney function describing factors. Third, plaque type cannot be analyzed by coronary angiography. Different plaque types, such as stabilized or vulnerable plaques, can affect miRNA plasma levels. Due to the lack of a full understanding of circulating miRNAs, more confounding factors remain unknown.

## 5. Conclusion

Plasma circulating miRNA-126 levels are increased in patients with severe atherosclerosis as determined by the SYNTAX score. Elevated miRNA-155 expression was observed in patients with Stage 1 eGFR but was lower in patients with Stages 2 and 3 eGFR. Plasma circulating miRNA-155 had positive correlations with higher levels of BMI, HOMA index, C-peptide, and triglycerides. The obtained data suggest that plasma circulating miRNAs might contribute to various pathological processes affecting the severity of CAD, kidney function, and metabolic syndrome. Despite various studies, the exact mechanisms and factors affecting miRNAs remain uncertain, and further studies are required.

### Funding

None.
